# Isolation of Garlic Bioactives by Pressurized Liquid and Subcritical Water Extraction

**DOI:** 10.3390/molecules28010369

**Published:** 2023-01-02

**Authors:** Marko Krstić, Nemanja Teslić, Perica Bošković, Darija Obradović, Zoran Zeković, Anita Milić, Branimir Pavlić

**Affiliations:** 1 AU “Julija Nova”, Save Mrkalja 26a, 11000 Belgrade, Serbia; 2 Faculty of Chemistry, University of Belgrade, Studenski Trg 16, 11000 Belgrade, Serbia; 3 Institute of Food Technology, University of Novi Sad, Bulevar Cara Lazara 1, 21000 Novi Sad, Serbia; 4 Department of Chemistry, Faculty of Science, 21000 Split, Croatia; 5 Institute of Physics Belgrade, University of Belgrade, Pregrevica 118, 11080 Belgrade, Serbia; 6 Faculty of Technology, University of Novi Sad, Bulevar Cara Lazara 1, 21000 Novi Sad, Serbia

**Keywords:** garlic, pressurized liquid extraction, subcritical water extraction, polyphenols, allicin

## Abstract

Garlic (*Allium sativum* L.) is widely used in various food products and traditional medicine. Besides unique taste and flavour, it is well known for its chemical profile and bioactive potential. The aim of this study was to apply subcritical water extraction (SWE) and pressurized liquid extraction (PLE) for the extraction of bioactive compounds from the Ranco genotype of garlic. Moreover, PLE process was optimized using response surface methodology (RSM) in order to determine effects and optimize ethanol concentration (45–75%), number of cycles (1–3), extraction time (1–3 min) and temperature (70–110 °C) for maximized total phenols content (TP) and antioxidant activity evaluated by various in vitro assays. Furthermore, temperature effect in SWE process on all responses was evaluated, while allicin content (AC), as a major organosulphur compound, was determined in all samples. Results indicated that PLE provided tremendous advantage over SWE in terms of improved yield and antioxidant activity of garlic extracts. Therefore, high-pressure processes could be used as clean and green procedures for the isolation of garlic bioactives.

## 1. Introduction

Garlic (*Allium sativum* L.) belongs to the Alliaceae family and has been cultivated around the world for centuries. It is widely used for culinary, but it is considered to be one of the most reliable disease-preventive foods with therapeutic characteristics. Garlic exhibits high biological activity, which is predominately attributed to sulphur compounds, particularly thiosulphates, alicin (AC) and phenolic compounds. These compounds are responsible for garlic medicinal properties, among which cardio-preventive and anti-hypertensive effects are the most common. Less known effects are related to reducing cholesterol levels in blood and helping patients with diabetes in glucose control [1,2]. With evolution of medicine and science, we are now able to extract those components and use them separately and in different applications.

The phenolic compounds have a significant bioactivity as an integral part of food. Therefore, it is necessary to have a simple, accurate, and green environmental technique for their analysis and extraction from plant material in food industry. Several studies have been conducted in relation to extraction of AC from garlic using solvent extraction, supercritical fluid extraction (SFE), ultrasonic-assisted extraction (UAE) and pressurized liquid extraction (PLE). The serious drawback of using solvent extraction is that extract might contain solvent with high toxicity and low purity [1,3,4]. These techniques are able to provide increased yield of target compounds from plant material and, at the same time, reduce energy consumption and number of operation units in separation and purification of crude extracts [1,3,4].

PLE is labelled as a green procedure, because solvents used in this technique (water and ethanol) could be natural, safe, non-toxic and also cheap processing fluids [5,6]. In certain cases, higher extraction yields of bioactive compounds can be obtained with methanol, but those extracts could not be consumed by humans due to the following side effects and toxicity [7]. Green procedure means that extract can be freely consumed by humans with no fear of side effects nor toxicity. In addition, both water and ethanol can be found in abundance in our environment and at very low cost, so the general procedure is also very economical [8,9]. PLE is, in general, a simple procedure with three main variables that can be adjusted specifically for every plant material and extraction process itself. Those variables are temperature, pressure and time of exposure (extraction). Changes in these parameters directly induce physical changes within applied solvent (water or ethanol) in the way that those compounds became more or less polar [10,11]. There is a general and scientifically proven rule that polar compounds are optimally dissolved in polar solvents, and vice-versa for non-polar compounds [12]. The idea of this method is to increase or decrease the polarity of the solvent to the just right amount in order to provide the maximum extraction of targeted compound from the extraction material [5].

Water has gained increasing attention as an extraction solvent due to its unique dissolving properties, which can be altered by changing the temperature [13]. Subcritical water extraction (SWE) is an extraction technique using water as the solvent, but with modified physical properties; it is considered a recent alternative for the isolation of antioxidant constituents from plant materials. The subcritical water is water at temperatures above its normal boiling point (100 °C) and below its critical point (374 °C) at a pressure at which it remains in the liquid state. At these conditions, water becomes less polar and therefore it is a suitable replacement for organic solvents [14]. At a temperature of above 374 °C and a pressure of above 220 bar, water is considered to be in the supercritical state [10]. SWE offers a suitable, safe, cost-effective and environmentally safe alternative compared to other methods as it takes advantage of non-polar compounds extraction [10].

A major disadvantage of SWE and PLE is the high operating pressure, which requires expensive equipment [15]. However, in the case of antioxidants, price should not play a limiting role, as natural antioxidants are desirable food components and costs are compensated by other advantages, such as the high purity of extracts and the efficiency of the process [16,17]. Due to the application of high temperatures, enhanced extraction of various organic compounds is allowed in SWE. In addition, the formation of various new compounds can be expected such as generation of new antioxidants [18]. This is possible through Millard and caramelization reactions forming 5-hydroxymethylfurfural (5-HMF) and 2-furaldehyde (F). As 5-HMF has been demonstrated to be cytotoxic in higher concentration, it is of great interest to gain an insight into the formation of this compound during SWE processes from natural matrices. In a case of SWE, according to the Tomšik et al. [9], no influence of 5-HMF and F on antioxidant activity was observed for extraction of bioactive compounds, especially antioxidants, from wild type of garlic (*A. ursinum*).

Moreover, the dielectric constant of subcritical water decreases to that of polar organic solvents with the increasing temperature [19], and it is further reduced by adding an organic solvent such as ethanol [20]. The change in the dielectric constant of the extractant would change the solubility of the extracted substances, which results in different compositions being dissolved. Thus, employing a subcritical treatment using aqueous ethanol or water on the same sample would yield different extract compositions [21]. In the literature, the subcritical aqueous ethanol treatment has been extensively used to extract carbohydrates and phenolic compounds [22,23,24,25,26,27,28].

An important fact is that not every garlic variety has the same amount and type of bioactive components, and therefore the efficacy of different types of garlic in medicinal use is not the same. Generally, AC is not naturally present in garlic, but rather synthetized from alliin by enzyme alliinase in the presence of water. In order to extract AC, the SWE can be used as it allows high purity of product. Water possesses a mimic of the properties of organic solvent and the enzymatic and extraction processes occur in one system [1].

Ranco genotype of garlic is considered to be very rich in bioactive components [29,30]. However, there are few scientific papers which are concerned with this specific genotype of garlic and PLE recovery of bioactive compounds from garlic. Therefore, the aim and novelty of this study was the application of PLE using aqueous ethanol and water as solvents for the extraction of bioactive compounds from the Ranco genotype of garlic. Moreover, PLE process was optimized using response surface methodology (RSM) in order to achieve simultaneous maximization of total phenols (TP) yield, allicin (AC) content, as well as maximized antioxidant activity (AOA) determined by DPPH· and ABTS^+^· radical scavenging assays, and ferric reducing power (FRAP) assay.

## 2. Results and Discussion

### 2.1. Subcritical Water Extraction

**Figure 1:**
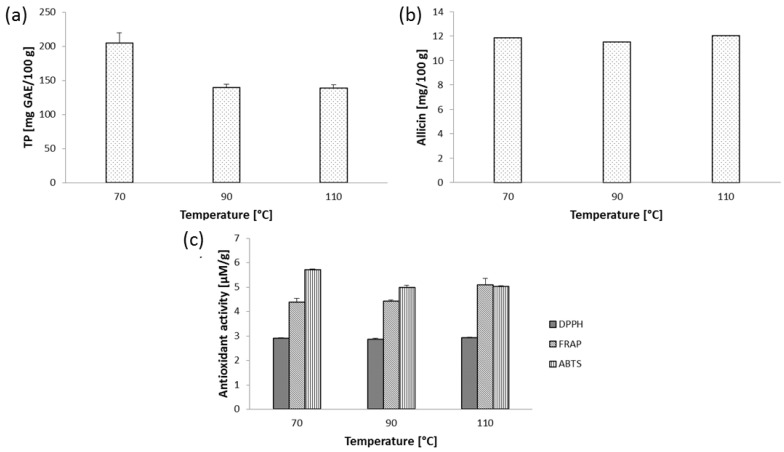
(**a**) TP, (**b**) allicin (AC) content and (**c**) antioxidant activity of *A. sativum* extracts obtained by SWE.

Subcritical water extraction (SWE) of *A. sativum* was performed at fixed set of cycles (two) and extraction time (2 min), while temperature was varied at three levels (70, 90 and 110 °C). Extracts were characterized in terms of TP, AC content and in vitro antioxidant activity determined by three assays (DPPH, FRAP and ABTS). Results are presented in Figure 1.

Experimentally observed TP in garlic extracts obtained by SWE was 204.80, 139.37 and 138.92 mg GAE/100 g at 70, 90 and 110 °C (Figure 1a). To the best of our knowledge, TP were not determined in garlic extracts obtained by SWE previously. Cavalcanti et al. [31] optimized solvent mixture and observed the highest TP (5.84 mg GAE/g) when polarity of the water was modified by the addition of ethanol and acetone at following volumetric ratio: water/ethanol/acetone, 4:1:1. Furthermore, TP observed in wild garlic (*Allium ursinum* L.) leaf extracts obtained by SWE varied from 0.971 to 4.002 g GAE/100 g depending on the applied temperature (120–200 °C), extraction time (10–30 min) and HCl concentration in water (0–1.5%) in static (batch-type) SWE extractor [9].

On the other hand, SWE temperature did not cause significant variation in AC yield due to very narrow range (11.56–12.08 mg/100 g) obtained at different temperatures (Figure 1b). According to Zaini et al. [1], various emerging extraction techniques, particularly high-pressure processes, could be useful for AC extraction as green alternative for conventional approach. In another work, Zaini et al. [32] compared alliin recovery from *A. sativum* by SWE and Soxhlet extraction. SWE was evaluated at two temperatures (120 and 180 °C) and solvent flow rates (2 and 6 mL/min) and results suggested that SWE improved recovery of alliin comparing to Soxhlet extraction, while the highest concentration of alliin (136.82 mg/g) was observed at 120 °C and 2 mL/min flow rate. Authors indicated that SWE could be efficiently used as green alternative for traditional extraction approach.

The model systems were used to evaluate in vitro antioxidant activity of garlic extracts obtained by SWE and PLE. Results suggested that temperature contributed to very small variations in antioxidant activity determined by DPPH test (2.88–2.92 µM TE/g). The highest capacity of garlic extracts toward scavenging of ABTS^+^ radicals (5.72 µM TE/g) was observed at the lowest temperature, while extract obtained at 110 °C contributed with the highest reducing power measured by FRAP assay (5.08 µM Fe^2+^/g). According to Ciric et al. [33], garlic extracts exhibited potent scavenging capacity toward DPPH radicals and reducing power determined by CUPRAC assay; however, results could not be compared since extraction was performed from the fresh sample and results were expressed as percent of inhibition for DPPH test. These authors also reported moderate correlation between polyphenol content and antioxidant activity. Tomsik et al. [9] determined that the optimal SWE conditions for simultaneous recovery of polyphenols and maximized antioxidant activity determined by DPPH and ABTS assays of wild garlic leaf extracts were a temperature of 179 °C, an extraction time of 10 min and 1.09% of acid modifier in water.

### 2.2. Pressurized-Liquid Extraction

#### 2.2.1. Model Adequacy

The face-centred central composite experimental design (CCD) was developed to evaluate impact of PLE variables (ethanol concentration, number of cycles, extraction time and temperature) on TP, AC and AOA (DPPH, FRAP, and ABTS tests) in garlic extracts (genotype Ranco). A total of 29 experimental runs were performed and experimental results of TP, AOA and AC of garlic extracts obtained with pressurized aqueous ethanol extraction under various process conditions are presented in Table 1.

The range of experimentally obtained TP was from 92.36 to 391.45 mg GAE/100 g, with the highest TP achieved when process parameters were set to a temperature of 110 °C, extraction time of 3 min, a number of cycles of 3 and ethanol concentration of 45% (run 16). The lowest value for TP was observed with run 4 which was set at the lowest applied temperature (70 °C), extraction time (1 min) and number of cycles (1) and the highest ethanol concentration (75%). Songsungkan and Chanthai [34] reported a somewhat lower TP content for garlic powder (362.5–542.2 μg GAE/g DW) and fresh garlic (831.5–1865.9 μg GAE/g DW) in comparison to the best performing PLE run (16; Table 1). This was probably caused by different extraction technique and raw material quality. Beato et al. [35] reported that TP of ten garlic cultivars cultivated in four different Spanish locations were in the range from 3.4 to 10.8 mg GAE/g DW, suggesting that climate, soil characteristics and garlic variety also have significant impact on TP. Extracts obtained with PLE (98.57–391.45 mg GAE/100 g) were generally superior to those obtained with SWE (110.32–204.80 mg GAE/100 g) in terms of TP, thus PLE was selected for further optimization process.

The range of experimentally obtained DPPH· radical scavenging activity was from 2.35 to 3.92 µmol TE/g, with the highest achieved at the following conditions: a temperature of 110 °C, extraction time of 3 min, a number of cycles of 1 and ethanol concentration of 75% (run 12), while the lowest value for DPPH assay was observed in experimental setup 4 (Table 1). As already mentioned, these particular extraction conditions resulted also in the lowest values for TP, as well as in very low values for FRAP, ABTS tests and AC. On the other hand, the highest TP and relatively high DPPH· scavenging activity, AC and ferric reducing power was observed for run 16 (Table 1). This indicates that phenols and AC have synergic effect on AOA in the obtained garlic extracts, as it was also reported by Songsungkan and Chanthai [34]. Experimentally obtained values of FRAP assay were from 4.54 to 10.17 µmol Fe^2+^/g, while for ABTS^+^, radical scavenging assays ranged from 4.66 to 7.63 µmol TE/g. Same as for DPPH test, the highest values of FRAP and ABTS assays were observed at run 12 (Table 1). Comparison of AOA with other studies related to garlic was hardly possible due to differences in assay protocols.

The highest AC (24.04 mg/100 g) was achieved at a temperature of 70 °C, extraction time of 1 min, a number of cycles of 3 and ethanol concentration of 75% (run 27; Table 1). The minimum concentration (16.03 mg/100 g) was obtained at the following conditions: ethanol concentration of 75%, number of cycles of 1, extraction time of 1 min, temperature of 110 °C. A different study related to PLE of AC from garlic reported comparable results (332 μg/g sample) to AC obtained in present study [36]. Differences between results could originate from various causes as it was already explained for TCP. Similarly to AOA and TP, AC was generally higher for PLE (Table 1) compared to SWE (Figure 1b), confirming a necessity to select PLE for optimization process. Besides polar solvents such as various aqueous alcoholic solutions, AC could be extracted with non-polar solvents such as supercritical CO_2_ (SCCO_2_). Del Valle et al. [37] reported that AC derived from garlic flakes obtained by SCCO_2_ was in the range of 0.031–0.176 mg/kg substrate, which is significantly lower than that of results achieved in present study. On the other hand, significantly higher AC content was determined in fresh white garlic (3.37–4.60 mg/g sample), elephant garlic (0.73 mg/g) and garlic powder extracts (2.26–3.05 mg/g) also isolated with SCCO_2_ [38], suggesting that AC could differ to a great extent even when same extraction techniques are utilized.

Experimental results for all individual responses were fitted to a second-order polynomial model (Equation (1)). The ANOVA results of fitting polynomic model of second degree are presented in the Table 2.

The descriptive statistics often do not provide sufficient data of the model adequacy; therefore, ANOVA, i.e., decision-making statistics, should be applied. Therefore, model adequacy was determined by Fisher’s test (F-test) for the model, coefficient of determination (R^2^), coefficient of variance (CV) and lack of fit. Firstly, quadratic models for AC and ABTS assay were not significant (*p* > 0.05), thus quadratic models of these responses were not considered. On the other hand, mathematical dependency between experimental data and the model equation was highlighted by significant *pm*-values (0.0001 ≤ *pm* < 0.02) (Table 2) [39]. Relatively high R^2^ for TP, DPPH· scavenging activity and ferric reducing power assay (0.898, 0.760 and 0.882, respectively), suggested that these three applied mathematical models were in accordance with obtained experimental results. Dispersion of the experimental data was evaluated by CV value. Since a small value of CV indicates low variation in the mean value, the relatively low CV obtained for DPPH test (8.72%) and moderate CV value for TP and FRAP assay (17.49 and 11.72%, respectively) suggested somewhat accurate reproducibility of the investigated quadratic models (Table 2) [40]. The lack of fit was insignificant (*pfit* > 0.05) for the three considered responses and there is a good agreement between predicted and experimentally obtained values [41].

#### 2.2.2. Impact of PLE Variables on Targeted Responses

Impact of 4 PLE variables was examined only for TP, FRAP and DPPH assays since these responses have significant quadratic models (Table 2). Linear term of ethanol concentration (ETC) exhibited a significant negative correlation with TP (Figure S1, Supplementary Materials). Considering ETC for recovery of polyphenols from various plant matrices, there is not a “golden rule” which is an optimal concentration. This is because apart from experimental conditions, polyphenol chemical structures and their interactions with matrix also play an important role in determination of TP. For example, phenolic content in present study displayed a decreasing trend from minimal to maximal applied ethanol concentration (45–75%) (Figure S1). In a recent study, it was reported that the highest TP in beet root extract was obtained for PLE coupled with ETC30%, then ETC70%, while the lowest was obtained when coupled with ETC50% and when other parameters were constant [27]. TP in black and red currant extracts was the highest when PLE was combined with ETC50%, ETC30% and ETC70%, respectively [28]. For wild thyme, the best performing conditions in terms of TP were with ETC30%, slightly lower with ETC60%, and the lowest with ETC45% [26]. Compared to ETC, linear term of temperature had a positive impact on TP (Table 2; Figure S1). Firstly, temperature influences molecular diffusivity and solvent viscosity [42], allowing better penetration of solvent at higher temperatures. Alterations in temperature in PLE are also followed with changes in solvent dielectric constant (DEC) which is a measure of polarity. Water and ethanol at ambient conditions have highly hydrogen-bonded structures, which are destroyed to an appreciable extent as the temperature is increased. This makes them less polar, and facilitates the dissolution of less polar substances and consequently influences TP. For example, DEC for ETC50% is 48.02, 34.71 and 26.00 at 40, 120 and 200 ° C, respectively [43]. Under these conditions, TP in *Stevia rebaudiana* Bertoni leaves was 53, 58 and 79 mg/g at the first, second and third experimental setup, suggesting that lower DEC which is achieved at higher temperatures would in certain cases lead to higher TP yield as it was also concluded in present study (Table 2; Figure S1). Mrkonjić et al. [26] also reported that with increase in temperature from 130 to 170 °C, there was almost a constant increase in TP in wild thyme extracts. The same conclusion was reported for recovery of phenols from avocado peel with ECT50% and temperature range from 40 to 200 °C as PLE parameters [44]. Positive impact of temperature on TP was also reported for *Laurus nobilis* L. leaf extracts isolated with PLE and aqueous ethanol [45]. As it is often the case when extractions are relatively short (1–3 min), extraction time is positively correlated with TP (Table 2; Figure S1). The simplest explanation for this is that prolonged contact of solvent with plant material would increase mass transfer of targeted bioactive compounds and its concentration in crude extracts. Extraction time also exhibited positive impact on phenolics content when these bioactive compounds were recovered from wild thyme [26]. However, when phenolic compounds were isolated with PLE from olive leaves, extraction time was negatively correlated with TP [46]. This suggested that in certain cases, prolonged exposure to high pressure and high temperatures which are commonly applied in PLE (100 MPa and above 100 °C) could cause degradation of thermally labile compounds. Besides linear terms, interaction between ETC and extraction time and ETC and temperature had significant impact on TP (Table 2). As it can be seen from Figure S1, higher TP is achieved when longer extraction time is combined with lower ETC and when higher temperatures are combined with lower ETC, which is aligned with the aforementioned conclusions.

As it was already mentioned, AOA in garlic crude extracts is influenced by the presence of polyphenols (Table 1). Thus, it is rather expected that AOA is somewhat following the trend of TP. In particular, like for TP, linear term of extraction time and temperature had a positive effect on DPPH· scavenging activity and ferric reducing power (Table 2; Figures S2 and S3), suggesting that higher temperatures and longer extraction time causes recovery of compounds with significant AOA. Similar conclusion was reported by Mrkonjić et al. [26] for wild thyme extracts. However, contrary to TP, linear term of ETC exhibited a positive impact on FRAP assay values, and interaction of ethanol and time positively influenced the DPPH assay values (Table 2). Such outcome again indicates that phenols are not the only drivers of AOA in crude garlic extracts, and that AC also has certain impact on AOA (Table 1). Thus, it is of paramount importance to optimize extraction maximization of all targeted responses that were successfully fitted to a second-order polynomial model.

#### 2.2.3. Multi-Response Optimization of PLE

**Figure 2:**
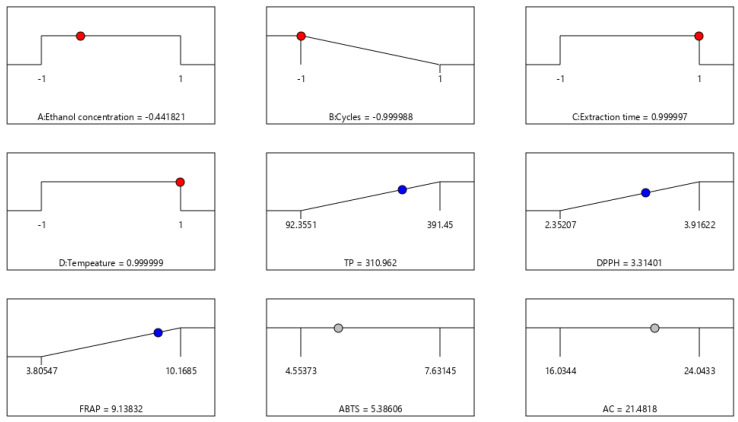
Optimal conditions for recovery of bioactive compounds from Ranco garlic genotype using PLE and predicted values of targeted responses.

TP and AOA (DPPH and FRAP tests) exhibited acceptable alignment with quadratic models, thus these responses were used as input for multi-response optimization of PLE. Regarding variables, the number of extraction cycles was fixed at the minimum (one cycle) since it did not significantly influence any of targeted responses (Table 2). This is beneficial from economic point of view as it would require less time, energy and solvent. The other three variables were set in the range of initial experimental design. Optimized PLE conditions for recovery of bioactive compounds from Ranco garlic genotype and predicted values at those conditions are presented in Figure 2.

PLE could be successfully utilized to obtain garlic extracts rich in polyphenols and AC which have significant antioxidant activity. Since extracts were obtained with GRAS (generally considered as safe) solvents (ethanol and water), they could be further used in food, pharmaceutical and nutraceutical industry.

## 3. Materials and Methods

### 3.1. Chemicals

1,1-Diphenyl-2-picryl-hydrazyl-hydrate(DPPH),2,2’-azino-bis(3-ethylbenzothiazoline-6-sulphonic acid) diammonium salt (ABTS·), Trolox 6-hydroxy-2,5,7,8-tetramethylchroma-2-carboxylic acid), Folin-Ciocalteu reagent and (±)-catechin were purchased from Sigma (Sigma-Aldrich GmbH, Sternheim, Germany).

Gallic acid was purchased from Sigma (St. Luis, MO, USA). Potassium persulfate (99% pure) was obtained from Acros Organics (Geel, Belgium). All other chemicals used were of analytical reagent grade.

### 3.2. Plant Material

Garlic (*Allium sativum* L.), genotype Ranco cultivated in Serbia as the autochthonous species was used in this study. All garlic samples were grown on experimental field “Rimski Sancevi” in Institute for Field and Vegetables (Novi Sad, Serbia). The samples were cultivated under controlled conditions, following the standard agronomical procedures at the Institute of Field and Vegetable Crops in Temerin (Novi Sad, Serbia). All samples of garlic were healthy without any sign of disease detected macroscopically.

### 3.3. Sample Preparation

Drying of the garlic sample was conducted according to a previously optimized procedure performed in a vacuum dryer [47]. Briefly, fresh garlic was peeled, chopped and let to rest for 30 min. The sample was further exposed to direct contact with heat surface and thus dried with both conduction and convection. Heat surface temperature, air pressure in vacuum chamber and the change in product weight during the drying process were controlled and registered by the drying procedure control system (PLC). Sample size was approximately 200 g of fresh garlic. Weight loss was recorded in 10 min intervals and drying was continued until no mass change was detected (final moisture content in equilibrium). Drying conditions were set in the following optimized order: surface temperature of 78 °C, air pressure of 30 mbar and drying time of 11 h. Moisture content in dried garlic sample was 4.79%.

### 3.4. Pressurized Liquid Extraction and Subcritical Water Extraction

Pressurized liquid extraction (PLE) with aqueous ethanol as solvent was performed in batch-type high-pressure extractor (ASE 350, Dionex, Sunnyvale, CA, USA) with maximum operating pressure of 100 bar and temperature of 200 °C. Auto-tuning for each temperature was performed by temperature controller before extraction; temperature regimes for each experimental run are presented in Table 1. Detailed extraction procedure was previously described elsewhere [26]. This experiment consisted of 4 variables (input parameters): ethanol concentration (45, 60 and 70%, *w*/*w*), number of extraction cycles (1, 2 and 3), temperature (70, 90 and 100 °C), and time of extraction per cycle (1, 2 and 3 min). For each experimental run, 5.0 g of plant material was mixed with 1.25 g diatomaceous earth. Rinsing volume was set at 30% while N_2_ purging time was set at 90 s. After extraction, extracts were immediately filtered through filter paper under vacuum (V-700, Büchi, Switzerland) and constituted up to 50 mL with solvent. Extracts were collected into glass flasks and stored at −20 °C until the analysis. In addition to 29 extractions according to the experimental plan with aqueous ethanol of different concentrations, 5 PLE with water were performed (samples 30–34, Table 1).

### 3.5. Total Phenols Content (TP)

Total phenols content (TP) in obtained extracts was determined using Folin–Ciocalteu procedure described in detail by Pavlic et al. [48]. Gallic acid was used as standard for preparation of calibration curve, and absorbance of the samples was measured at 750 nm (6300 Spectrophotometer, Jenway, UK). The content of phenolic compounds was expressed as mg of gallic acid equivalents (GAE) per 100 g of sample. All experiments were performed in triplicate, and results are expressed as mean values.

### 3.6. DPPH· Scavenging Assay

Free radical scavenging activity of samples was determined using DPPH· assay, previously described by Pavlic et al. [48]. Solution of DPPH· reagent in methanol (65 µmol) was freshly prepared and adjusted with methanol to achieve absorbance of 0.70 (±0.02). A total of 2.9 mL of DPPH· reagent and 0.1 mL of properly diluted extracts were mixed and incubated for 60 min at ambient temperature. Absorbance was measured in three replicates at 517 nm (6300 Spectrophotometer, Jenway, UK). Calibration curve was obtained by measuring free radical scavenging of freshly prepared Trolox aqueous solutions (0–0.8 mM). The obtained results were reported as µmol of Trolox equivalents per g of dry weight (µmol TE/g). All experiments were performed in triplicate, and results are expressed as mean values.

### 3.7. Ferric Reducing Antioxidant Power (FRAP) Assay

Reducing power of the samples was determined according to assay based on the reduction of Fe^3+^ by polyphenol antioxidants, previously described by Pavlic et al. [48]. The FRAP reagent was freshly prepared from 300 mM acetate buffer (pH = 3.6), 10 mM 2,4,6-tris(2-pyridil)-s triazine (TPZT), 40 mM HCl solution and 20 mM FeCl_3_ aqueous solution, which were mixed in a 10:1:1 (*v*/*v*/*v*) ratio. A total of 1.9 mL of FRAP reagent and 0.1 mL of properly diluted extracts were mixed and incubated at 37 °C in the dark for 10 min. Absorbance was measured in three replicates at 593 nm (6300 Spectrophotometer, Jenway, UK). Calibration was performed using freshly prepared Fe^2+^ (Fe_2_SO_4_) aqueous solutions (0–0.23 mM). The obtained results were reported as µmol of Fe^2+^ equivalents per g of sample (µmol Fe^2+^/g). All experiments were performed in triplicate, and results are expressed as mean values.

### 3.8. ABTS^+^· Scavenging Assay

The ability of garlic extracts to neutralize ABTS^+^ radicals was determined by spectrophotometric method previously described by Pavlic et al. [48]. The ABTS reagent solution was prepared by combining and stirring of 7 mM aqueous solution of a 2,2′-azino-bis (3-ethylbenzothiazoline-6-sulfonic acid) diammonium salt (ABTS) and 2.45 mM potassium persulfate in a ratio of 1:1 (*v*/*v*). The incubation was performed at room temperature for 16 h in the dark. ABTS reagent was diluted with acetate buffer (pH 3.6) to adjust the absorbance at 734 nm to 0.70 ± 0.02. Appropriate dilutions of liquid extracts (0.1 mL) were mixed with ABTS reagent (2.9 mL) and incubation was performed at room temperature in the dark for 300 min. The absorbance measurement was performed at 734 nm and in order to obtain a calibration curve, freshly prepared aqueous Trolox solution (0–0.8 mM, R^2^ = 0.973) was used. In the case of liquid extracts, the results are expressed in mM equivalent of Trolox per g of dry garlic sample (µmol TE/g).

### 3.9. Allicin Determination by LC-MS/MS

Garlic extracts were quantitatively transferred into PP tubes of 50 mL and centrifuged in the centrifugation machine (MPW-56, MPW Med. Instruments, Poland) at 3000 rpm during 5 min. The supernatant (1 mL) was filtered through membrane filter (0.45 µm) into the glass vial. In this way, all samples were prepared for determination of the AC content with new LC-MS/MS method, along with the UV detector using device System Triple Quad 6500+ LC-MS/MS. The flow rate was set to 0.25 mL/min, and UV detection was performed at 240 nm. The optimal separation characteristics were achieved by using the Endcapped C18 Aq HPLC column (3 μm, 2.1 × 100 mm, United Chemical Technologies, Bristol, PA, USA), and a mixture of acetonitrile (A) and water acidified with 0.1% *v*/*v* formic acid (B) as the mobile phase. Table S1 shows the gradient conditions used for the determination of AC content. The LC–MS/MS method was validated with respect to the specificity, linearity (*r* = 0.9999), sensitivity, precision (*RSD* = 1.30%), accuracy, and recovery (98%). The obtained limit of detection (LOD) was 0.5 ng/mL, while the obtained limit of quantification (LOQ) for AC was 2 ng/mL. The content of AC in extracts was expressed as mg/100 g of garlic.

### 3.10. Experimental Design and Statistical Analysis

The CCD with 4 independent variables on 3 levels was applied, whereby 29 extractions were performed. The effects of input factors (ethanol concentration, temperature, number of cycles and extraction time per cycle) on targeted responses (AC, TP, DPPH· scavenging activity, FRAP, and ABTS^+^· scavenging activity) were investigated. Experimental plan with details regarding input factors and experimentally obtained values for investigated responses are presented in Table 1. The response surface methodology (RSM) was applied to evaluate the effects of extraction parameters and to optimize conditions for various responses. The response variables were fitted to the following second-order polynomial model (Equation (1)):

(1) $$Y=\beta_{0}+\overset{4}{\underset{i=1}{\sum }}\beta_{i}X_{i}+\overset{4}{\underset{i=1}{\sum }}\beta_{\mathrm{ii}}X_{i}^{2}+\sum \overset{4}{\underset{i<j=1}{\sum }}\beta_{\mathrm{ij}}X_{i}X_{j},\;$$

where Y represents the response variable, X_i_ and X_j_ are the independent variables affecting the response, and β_0_, β_i_, β_ii_ and β_ij_ are the regression coefficients for intercept, linear, quadratic and interaction terms, respectively. The experimental design and multiple linear regression analysis were performed using Design-Expert v.12 (Stat-Ease, Minneapolis, MN, USA). The results were statistically tested by analysis of variance (ANOVA) with the significance level of 0.05. The relationship between the response and independent variables was demonstrated using 2D graphics.

## 4. Conclusions

The application of SWE is an economical alternative to the conventional extraction methods due to the fact that for efficient extraction, shorter extraction time need to be applied, that this technology used widely available water as extraction solvent, and due to the possibility to directly and without further process of separation or purification use obtained extracts as semi-products or products for food or pharmaceutical industry. The obtained results show that higher temperature (110 °C) increases polyphenol extraction, and higher number of cycles increases antioxidant activity and AC concentration. The PLE method presents a unique possibility to extract a large number of wanted compounds from plants using only “green” procedures. Results show that a combination of a 53.4% ethanol, one cycle, a 3 min cycle time, and a 110 °C temperature results in the most optimal way to run subcritical ethanol extractions from Ranco genotype garlic obtained from the territory of Serbia and grown under controlled conditions which guarantee the maximum concentration of TP and AC and antioxidant activity of the extract. This study poses the question of introducing pressure and/or some chemical adjuvant in the described extraction method for the sake of promoting and improving the entire process.

## Figures and Tables

**Table 1:** Central composite experimental design with natural and coded values of independent variables and experimentally obtained values for investigated responses.

Run	Factor 1		Factor 2		Factor 3		Factor 4		Responses				
	Ethanol Concentration [%]		Cycles		Extraction Time [min]		Temperature [°C]		TP [mg GAE/100 g]	DPPH [µmol TE/g]	FRAP [µmol Fe^2+^/g]	ABTS [µmol TE/g]	AC [mg/100 g]
1	0	60	1	3	0	2	0	90	173.96	2.75	6.14	5.93	21.40
2	0	60	0	2	0	2	0	90	172.24	2.74	6.65	5.69	23.18
3	0	60	0	2	0	2	0	90	164.90	2.81	6.30	5.88	22.13
4	1	75	−1	1	−1	1	−1	70	92.36	2.35	4.54	4.66	17.66
5	1	75	1	3	−1	1	1	110	153.75	2.72	6.81	6.06	21.76
6	−1	45	1	3	−1	1	−1	70	146.33	2.52	4.83	4.94	21.41
7	1	75	−1	1	−1	1	1	110	149.63	2.85	7.02	6.55	16.03
8	−1	45	1	3	1	3	1	110	353.71	3.28	8.06	7.38	19.33
9	1	75	−1	1	1	3	1	110	130.54	3.12	8.95	5.99	20.09
10	0	60	0	2	0	2	1	110	284.16	3.41	8.64	7.06	17.71
11	0	60	0	2	−1	1	0	90	146.78	2.50	5.53	5.18	19.60
12	1	75	1	3	1	3	1	110	194.77	3.92	10.17	7.63	21.49
13	0	60	0	2	0	2	−1	70	118.56	2.49	5.09	4.87	21.79
14	0	60	0	2	0	2	0	90	181.52	3.15	7.53	4.87	22.09
15	−1	45	1	3	1	3	−1	70	136.98	2.51	4.99	6.12	19.09
16	−1	45	−1	1	1	3	1	110	391.45	3.44	8.61	5.02	21.29
17	0	60	0	2	0	2	0	90	198.59	3.14	7.50	6.50	22.18
18	0	60	0	2	1	3	0	90	172.61	2.92	7.47	6.15	20.53
19	−1	45	−1	1	1	3	−1	70	194.32	2.97	5.88	5.89	21.66
20	1	75	−1	1	1	3	−1	70	114.37	3.07	7.04	5.99	23.71
21	−1	45	−1	1	−1	1	1	110	200.87	2.53	4.53	5.50	22.08
22	0	60	0	2	0	2	0	90	154.27	2.79	5.49	4.98	23.11
23	0	60	−1	1	0	2	0	90	158.69	2.99	6.20	5.62	19.11
24	1	75	1	3	1	3	−1	70	141.32	3.38	7.54	5.91	21.08
25	−1	45	0	2	0	2	0	90	184.74	2.81	4.64	5.97	18.33
26	−1	45	−1	1	−1	1	−1	70	130.24	2.71	3.81	5.50	17.47
27	1	75	1	3	−1	1	−1	70	98.57	2.37	4.59	4.74	24.04
28	−1	45	1	3	−1	1	1	110	265.75	3.13	5.99	4.55	20.80
29	1	75	0	2	0	2	0	90	139.52	2.76	6.30	6.68	21.54

Presented results are mean values of three replicates (*n* = 3).

**Table 2:** The results of ANOVA analysis.

Term	TP	DPPH	FRAP
β0	172.08	2.87	6.46
**Linear**			
β1	−43.86 *	0.0358	0.6456 *
β2	5.70	0.0313	0.1417
β3	24.77 *	0.2734 *	1.17 *
β4	52.87 *	0.2241 *	1.14 *
**Interaction**			
β12	7.23	0.0749	0.0333
β13	−15.41 **	0.1184 **	0.1483
β14	−26.36 *	−0.0143	0.0975
β23	−5.95	0.0126	−0.1294
β24	6.47	0.0894	0.0778
β34	11.31	0.0331	0.2338
**Quadratic**			
β11	−8.09	−0.0283	−0.7856
β22	−3.90	0.0545	−0.0887
β33	−10.52	−0.1041	0.2439
β44	31.14	0.1348	0.6092
R^2 a^	0.898	0.760	0.882
CV ^b^	17.49	8.73	11.72
*pm*—Value ^c^	0.0001	0.0196	0.0003
*pfit*—Value ^d^	0.0847	0.3063	0.7177

* *p* < 0.01. ** 0.05 ≤ *p* < 0.1. ^a^ Determination coefficient. ^b^ Coefficient of variance (%). ^c^ Probability of F-value for the model. ^d^ Probability of F-value for the lack of fit.

## Data Availability

Data are contained within the article or Supplementary Material.

## Supplementary Materials

The following supporting information can be downloaded at: https://www.mdpi.com/article/10.3390/molecules28010369/s1, Figure S1: Influence of pressurized liquid extraction parameters on total polyphenol content in garlic extracts; Figure S2: Influence of pressurized liquid extraction parameters on DPPH· scavenging activity in garlic extracts; Figure S3; Influence of pressurized liquid extraction parameters on ferric reducing power in garlic extracts; Table S1: Gradient conditions for determination of AC content.

## Sample Availability

Data are contained within the article or Supplementary Material.
