# Supplementary material for: Isolation of Garlic Bioactives by Pressurized Liquid and Subcritical Water Extraction
Source: Molecules. 2023 Jan 2;28(1):369. doi: 10.3390/molecules28010369 (PMC9822463; doi:10.3390/molecules28010369)
Supplement: Supplementary file 1 [file molecules-28-00369-s001.zip › molecules-2105718-supplementary.pdf]

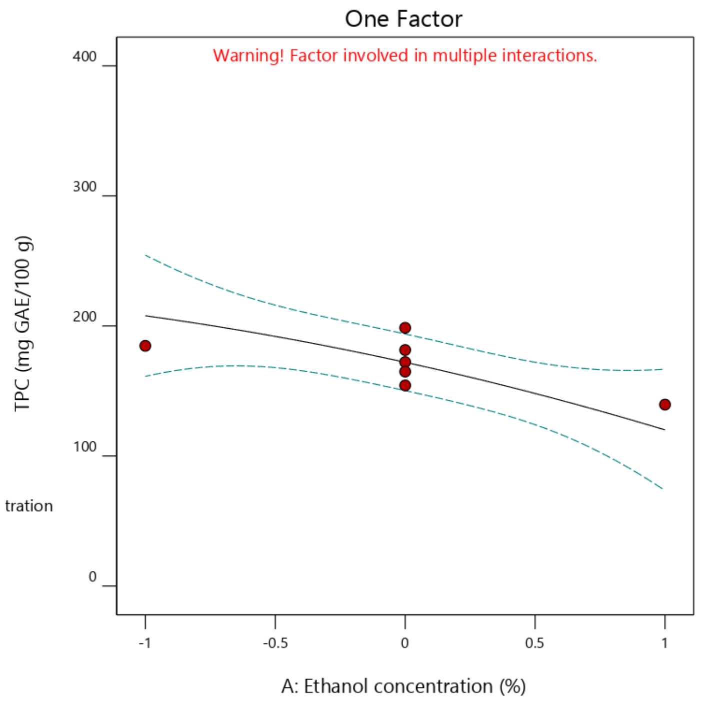
One Factor
Warning! Factor involved in multiple interactions.
400
300
200
100
0
TPC (mg GAE/100 g)
tration
-1
-0.5
0
0.5
1
A: Ethanol concentration (%)

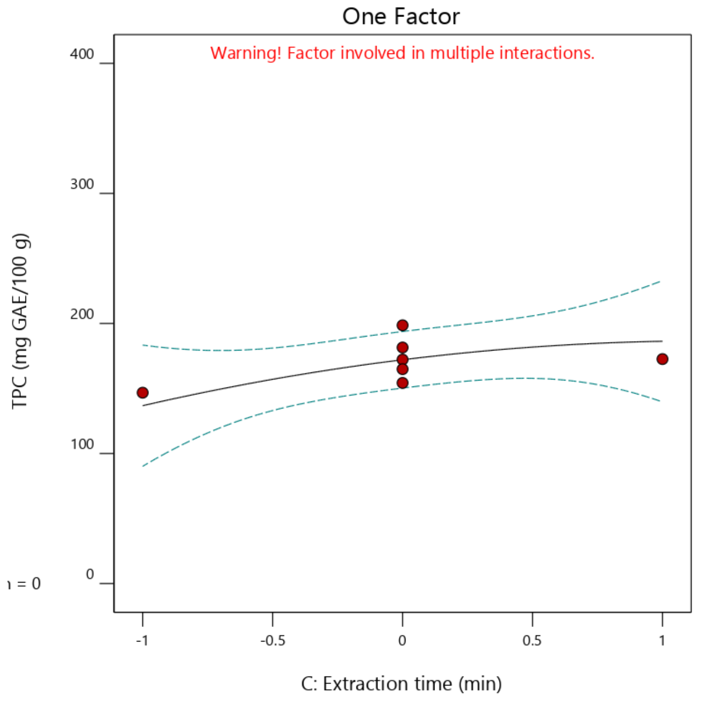
One Factor
Warning! Factor involved in multiple interactions.
400
300
200
100
0
TPC (mg GAE/100 g)
-1
-0.5
0
0.5
1
C: Extraction time (min)

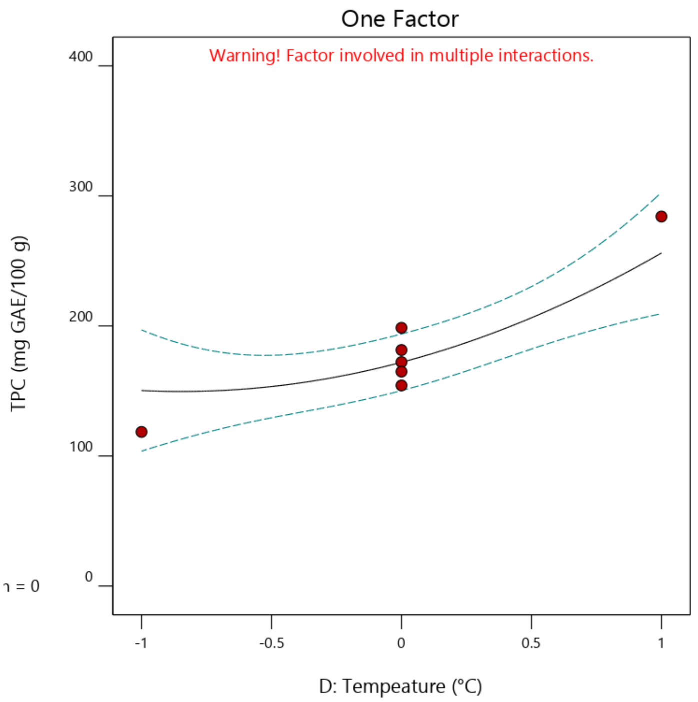
One Factor
Warning! Factor involved in multiple interactions.
TPC (mg GAE/100 g)
400
300
200
100
0
-1
-0.5
0
0.5
1
D: Tempeature (°C)

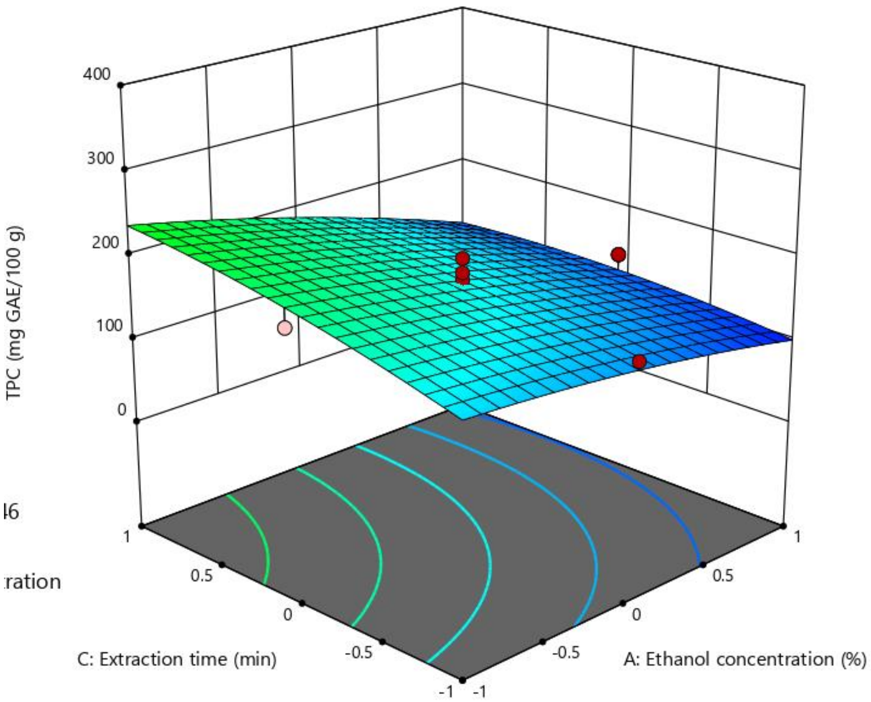
TPC (mg GAE/100 g)
400
300
200
100
0
C: Extraction time (min)
A: Ethanol concentration (%)
1
0.5
0
-0.5
-1

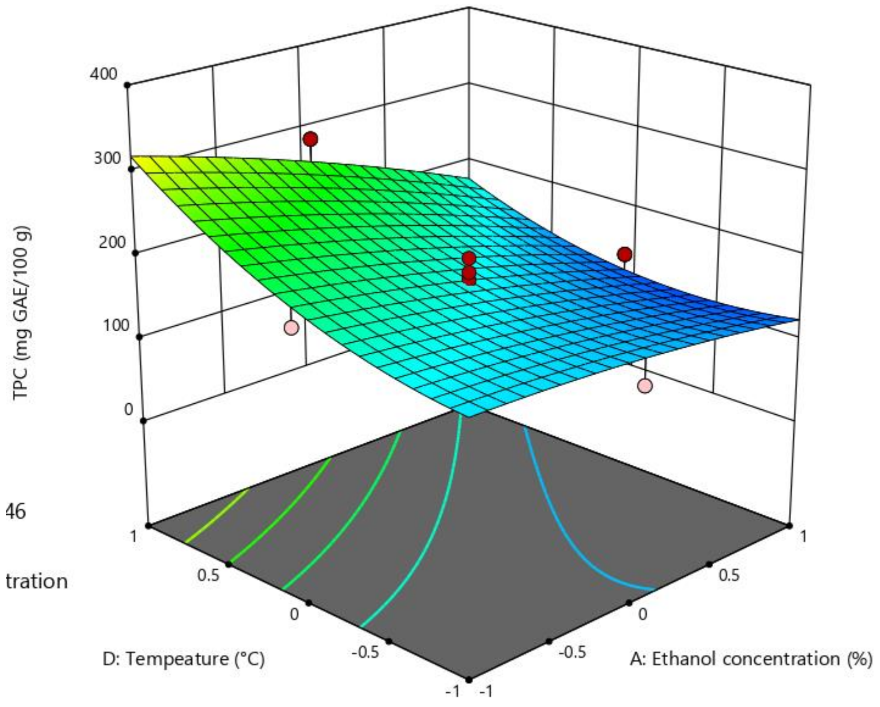

Figure S1. Influence of pressurized liquid extraction parameters on total polyphenol content in garlic extracts

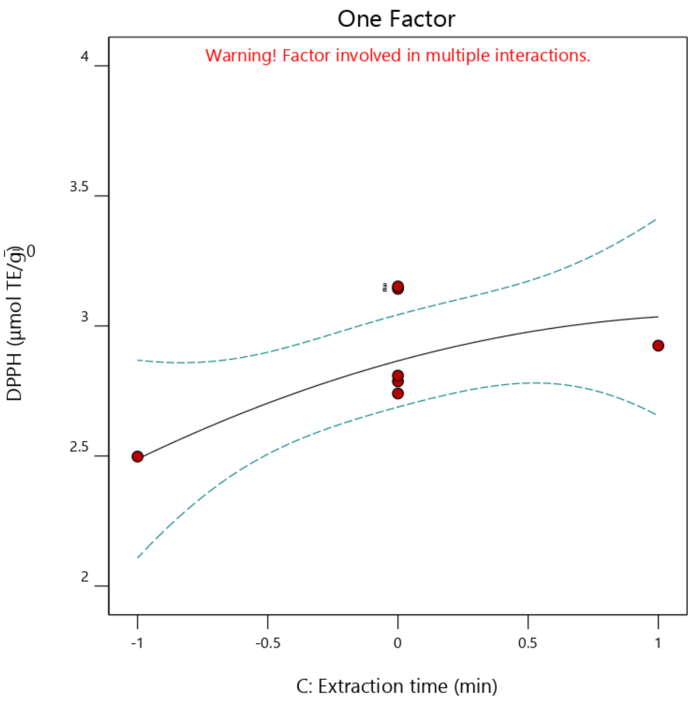
One Factor
Warning! Factor involved in multiple interactions.
DPPH (µmol TE/g)
4
3.5
3
2.5
2
-1
-0.5
0
0.5
1
C: Extraction time (min)

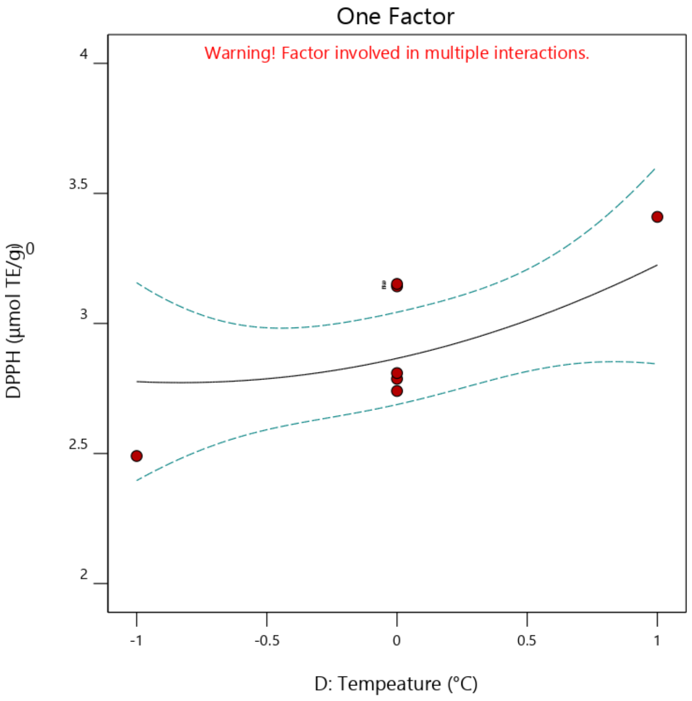
One Factor
Warning! Factor involved in multiple interactions.
DPPH (µmol TE/g)
4
3.5
3
2.5
2
-1
-0.5
0
0.5
1
D: Tempeature (°C)

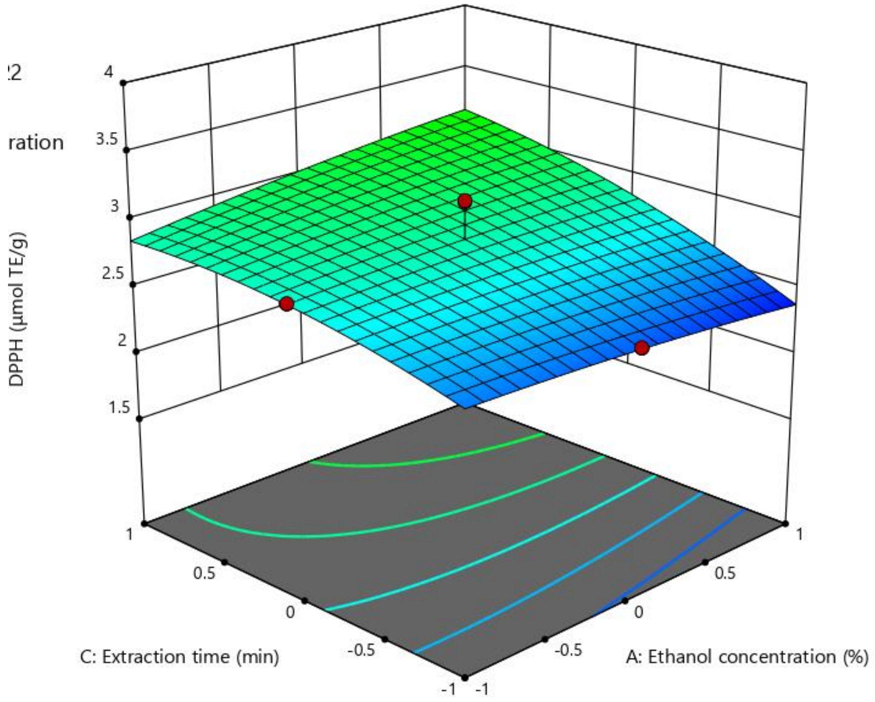

Figure S2. Influence of pressurized liquid extraction parameters on DPPH· scavenging activity in garlic extracts

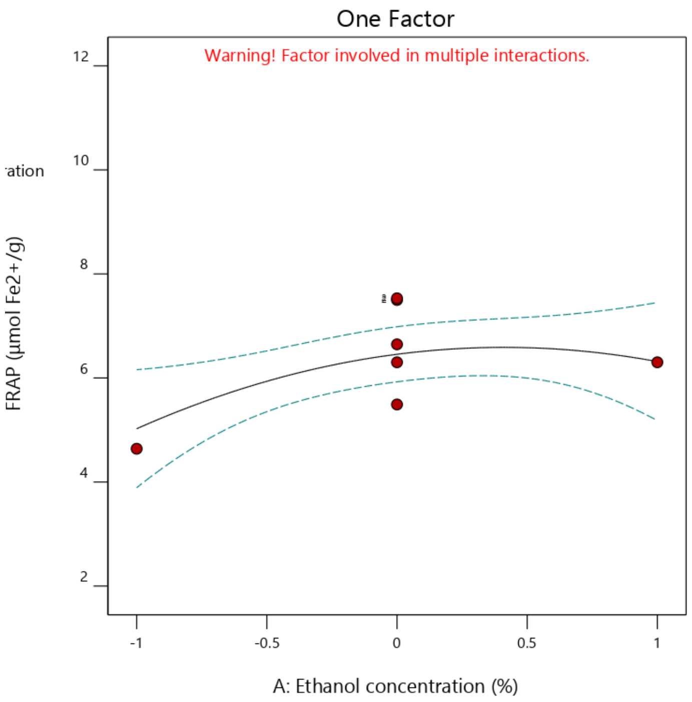
One Factor
Warning! Factor involved in multiple interactions.
FRAP (µmol Fe2+/g)
A: Ethanol concentration (%)
12
10
8
6
4
2
-1
-0.5
0
0.5
1

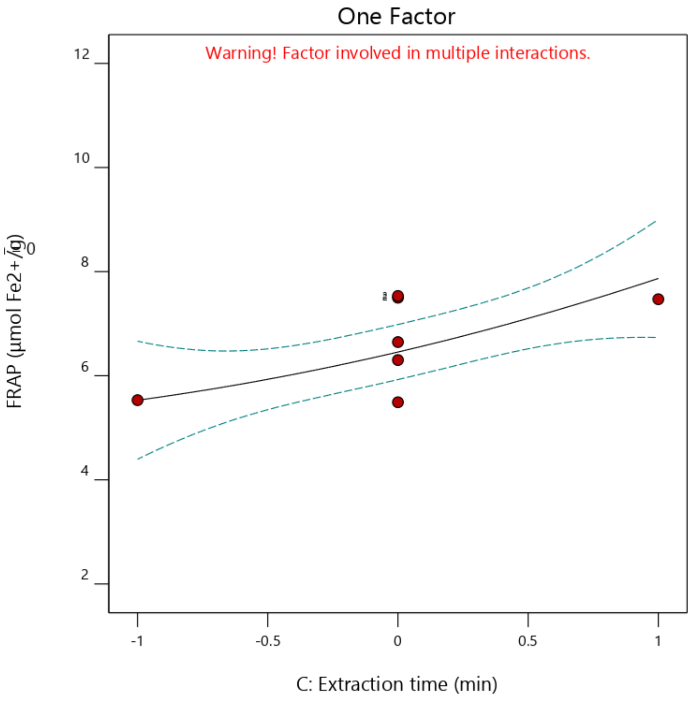
One Factor
Warning! Factor involved in multiple interactions.
FRAP (µmol Fe2+/g)
C: Extraction time (min)
12
10
8
6
4
2
-1
-0.5
0
0.5
1

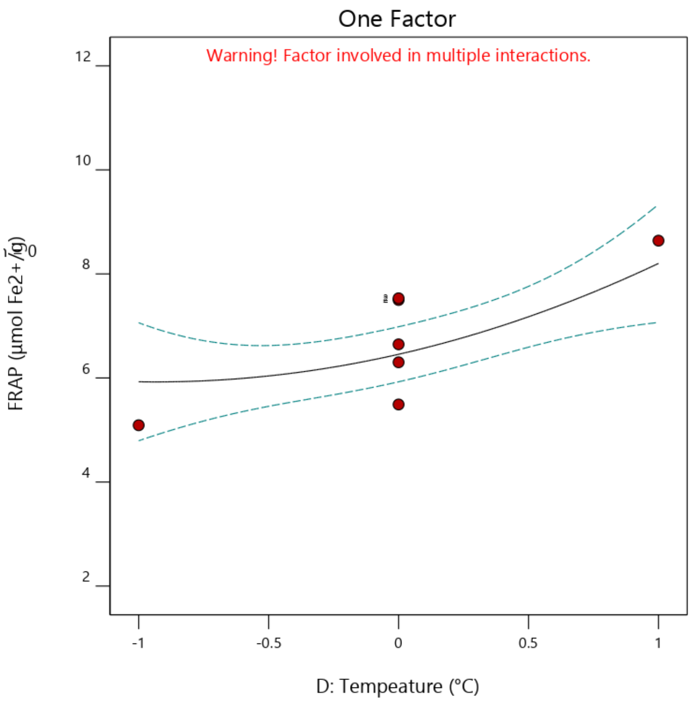

Figure S3. Influence of pressurized liquid extraction parameters on ferric reducing power in garlic extracts

Table S1. Gradient conditions for determination of AC content

**Time (min)**	**% B**
0.01	20
0.50	20
10.00	98
11.50	98
12.50	20
15.50	Stop
